# Biomimetic Microchannel Integrated Silk Fibroin Scaffold for Regeneration of Intervertebral Disc Degeneration

**DOI:** 10.34133/bmr.0203

**Published:** 2025-05-28

**Authors:** Tongxing Zhang, Zhaojun Cheng, Zhen Zhang, Lilong Du, Zhenhua Li, Zhuyan Jiang, Zhaomin Zheng, Deling Kong, Meifeng Zhu, Wen Li, Baoshan Xu

**Affiliations:** ^1^ Department of Minimally Invasive Spine Surgery, Tianjin Hospital, Tianjin University, Tianjin 300211, China.; ^2^ Department of Spine Surgery, The Second Affiliated Hospital of Guangzhou Medical University, Guangzhou 510260, China.; ^3^ Academy of Medical Engineering and Translational Medicine, Tianjin University, Tianjin 300072, China.; ^4^ Department of Spine Surgery, The First Affiliated Hospital, Sun Yat-Sen University, Guangzhou 510080, China.; ^5^ College of Life Sciences, Nankai University, Tianjin 300071, China.; ^6^ School of Disaster and Emergency Medicine, Tianjin University, Tianjin 300072, China.

## Abstract

Intervertebral disc degeneration (IVDD) is the primary cause of low back pain, and patients with severe degeneration usually require lumbar fusion or total disc arthroplasty. Lumbar fusion carries the risk of accelerated degeneration of the adjacent intervertebral disc (IVD), and total disc arthroplasty could reduce the risk. However, the clinical application of artificial IVD whose nondegradable properties make it difficult to restore the biological function of the IVD. Therefore, we intend to fabricate a novel biomimetic microchannel integrated silk fibroin scaffold (BMI-SF scaffold) containing annulus fibrosus with oriented cross-microchannels and nucleus pulposus with interconnected porous structure. The BMI-SF scaffold exhibits controllable microchannels as well as excellent biocompatibility and biodegradability. In vitro and in vivo studies have demonstrated that microchannels can direct cells into the BMI-SF scaffold and enhance neovascularization, supplying adequate nutritional support for tissue regeneration. The IVD replacement model showed that the BMI-SF scaffold has superior regenerative effects, such as restoring IVD height and providing motion segments with dynamic mechanical properties akin to the natural IVD. In this study, the BMI-SF scaffold developed using controlled microchannels provides a new strategy for patients with severe IVDD and has broad clinical application prospects.

## Introduction

Intervertebral disc degeneration (IVDD) is the primary etiology of low back and neck pain, leading to marked patient discomfort and imposing substantial economic costs on both families and society [1,2]. The current therapeutic approaches for IVDD are mainly conservative treatment and operative treatment. Patients with severe IVDD usually opt for operative treatment because conservative treatment fails. Current operative strategies for treating severe IVDD mainly include discectomy, spinal fusion, and total disc arthroplasty. Among them, total disc arthroplasty was once the preferred surgical method for clinicians because it could restore the motor function of the motion segment [2,3]. However, with the prolongation of postoperative follow-up, postoperative complications such as infections and graft displacement have been frequently reported. This is why the clinical application of artificial intervertebral disc (IVD) has become increasingly rare in recent years [4–6]. The main reason for the failure of the long-term therapeutic effect of artificial IVD was that the widely used artificial IVD was made of metals or polymers that were not degradable, making it difficult to integrate well with the adjacent tissue. The biological function of the motion segments cannot be restored. Tissue engineering technology has evolved as an innovative approach to the creation of artificial IVD.

Advancements in tissue engineering technology have led to various strategies aimed at creating artificial IVD scaffolds that replicate the hierarchical structure and function of real IVDs. The annulus fibrosus (AF) consists of several layers of collagen fiber lamellae organized in an orientated fashion, with collagen fibers intersecting between adjacent layers at an angle of ±30°. Reconstructing this complex hierarchical structure affects the mechanical properties and cell alignment of the AF scaffold [7]. Electrospinning technology is one of the important methods to solve the complex hierarchical design of AF [8]. Early research typically utilized electrospinning technology to produce aligned nanofiber membranes, which were then concentrically stacked or wrapped to form an AF scaffold, representing to some extent a biomimetic reconstruction of the hierarchical structure of the native AF [9,10]. However, the nanofiber membranes obtained by the electrospinning technique are unfavorable for cell infiltration due to their low porosity. Polymers can also be processed into oriented microfiber sheets using wet spinning technology [11,12]. Although the wet spinning technique can be used to produce an oriented fiber scaffold, there are limitations in the biomimetic reconstruction of the hierarchical structure of the native AF due to the lack of regulation of the fiber crossing angle. Our previous study found that melt spinning technology offers unique advantages in constructing of the biomimetic AF scaffold, mainly including precise and controllable fiber diameter and fiber crossing angle as well as customization of scaffold size. The AF scaffold fabricated using this technology has effectively repaired box-shaped AF defects in goats [13]. Nucleus pulposus (NP) is hydrated gel-like tissue, and water loss in the NP is considered the main pathological manifestation of IVDD [14,15]. Hydrogels have a similar gel morphology to NP and are rich in water content. Therefore, various hydrogels have been developed for NP regeneration [16–19]. The intricate structure of the IVD, comprising the AF and NP, has been identified. It is difficult to achieve ideal repair results for severe IVDD by simply repairing the AF or NP. In recent years, development work on total artificial IVD with biomimetic AF and NP has been conducted and some progress has been made [20–24]. In these studies, biomimetic reconstruction of AF and NP tissues was performed separately and then assembled into a total artificial IVD scaffold. Due to the complexity of the manufacturing process and the neglect of the integration of AF and NP, there are currently no reports on the clinical application of biomimetic artificial IVD that could overcome these problems. Three-dimensional (3D) printing technology has been demonstrated in the construction of artificial IVD scaffolds [24,25]. Due to the poor plasticity of natural biomaterials, polymer materials are currently widely used in the 3D printing process. Therefore, the process of using natural biomaterials for biomimetic construction of artificial IVD scaffolds still poses considerable challenges. In this study, we have innovatively used the reverse pore-forming technology to construct artificial IVD scaffolds with microchannel structures.

Tissue function depends critically on the arrangement of cells and tissues. Numerous studies have reported that microchannel scaffolds can guide aligned cell growth, regulate cell phenotype, and achieve functional tissue repair [26–29]. In our preliminary work, melt spinning technology was used to fabricate a biomimetic AF scaffold with oriented cross fibers. We changed the idea and used it as a sacrificial template for constructing microchannels in the AF area by reverse pore-forming technology. Additionally, the paraffin spheres created by phase separation technology were used as pore-forming templates to fabricate a porous scaffold featuring interconnected pore structures. This intricately linked porous scaffold is advantageous for tissue regeneration [30,31]. Likewise, we believe that paraffin spheres can be used as sacrificial templates for constructing porous microchannels in the NP area.

**Fig. 1.**
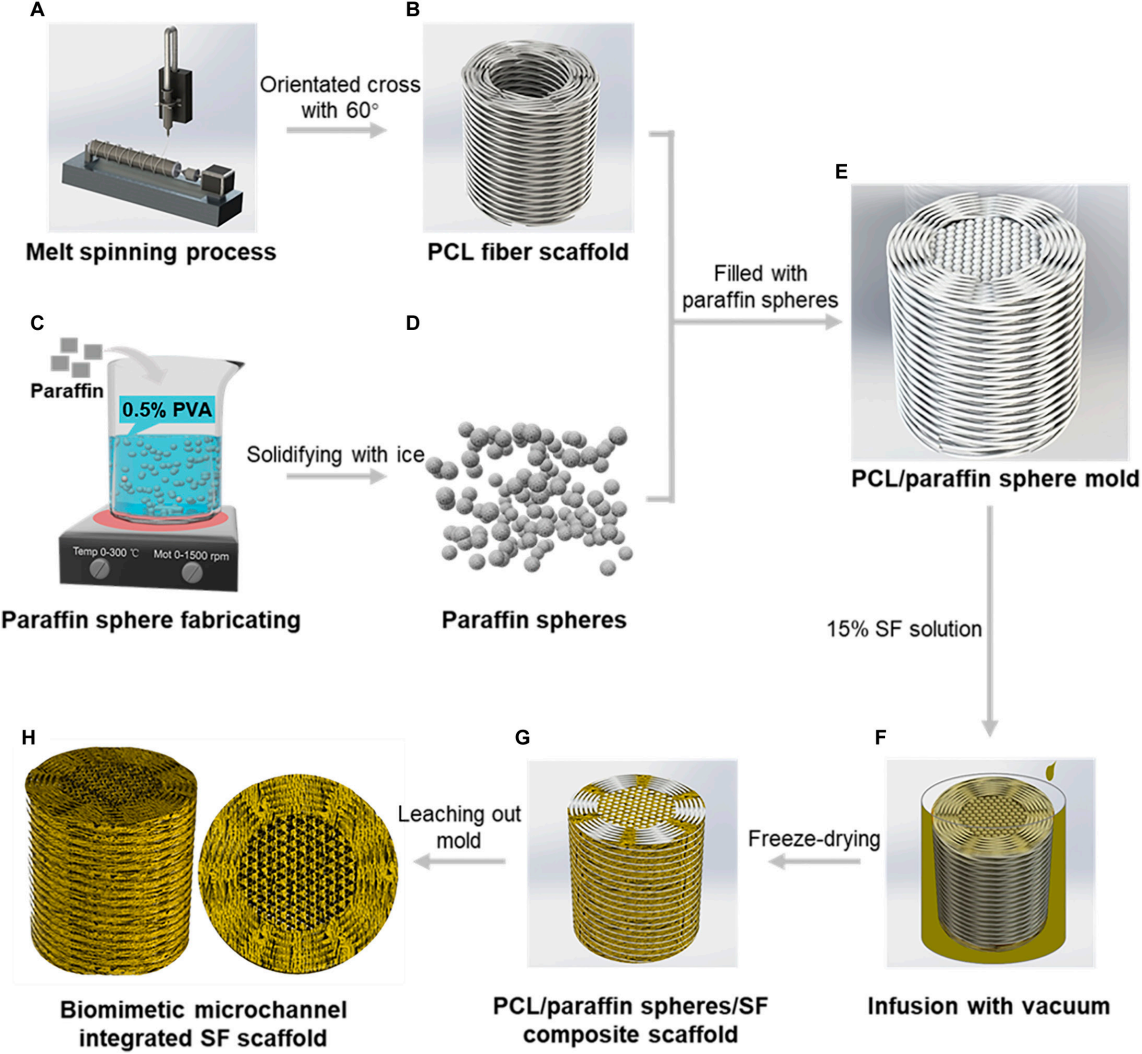
Schematic illustration of the procedure for fabrication of the BMI-SF scaffold. (A) Melt spinning process. (B) PCL fiber scaffold with orientated cross of 60°. (C) Paraffin spheres fabricated by lotion dispersion. (D) Paraffin spheres. (E) PCL/paraffin sphere mold. (F) Infusion of SF solution with vacuum. (G) PCL/paraffin/SF composite scaffold. (H) Biomimetic microchannel integrated SF scaffold.

This work aimed to create an innovative BMI-SF scaffold to facilitate the regeneration of severe IVDD by utilizing microchannels to direct cellular behavior and tissue infiltration. In summary, we first assembled the oriented cross polycaprolactone (PCL) fiber scaffold with paraffin spheres to form a complete sacrificial template, and the dimensions of sacrificial template were designed based on the caudal IVD of the rats weighing 300 to 350 g. In the recent work, we have completed the first step of the BMI-SF scaffold preparation. The ideal scaffold material should have good biocompatibility and appropriate biodegradability, and its degradation rate should be synchronized with the tissue regeneration process to achieve migration replacement of the scaffold material through the neotissues during tissue regeneration. Silk fibroin (SF) [32], as Food and Drug Administration (FDA)-approved biodegradable natural biomaterial, has outstanding advantages such as good biocompatibility, suitable mechanical properties, low immunogenicity, and abundant sources. It is commonly used in building bones, cartilage, and ligaments [33–35]. Given this background, SF was selected as the scaffold material for the second step of the BMI-SF scaffold fabrication, in which SF is infused into the sacrificial template and freeze-dried and the sacrificial template is leached out to obtain the BMI-SF scaffold (Fig. 1). In vitro and in vivo investigations were performed to assess the biomimetic structure, biocompatibility, capacity to direct cell behavior and tissue infiltration, integration with surrounding tissues, and biomechanical functional restoration of the BMI-SF scaffold. In this study, we innovatively utilized reverse pore formation technology to introduce microchannel structures into the fabrication process of artificial IVD scaffold, and we have attempted to control the behavior of cells and the deposition and arrangement of extracellular matrix by microchannel structures, providing new ideas for a quasi-natural regeneration of IVD.

## Materials and Methods

### Materials

PCL was purchased from Sigma-Aldrich (St. Louis, MO, USA). Mulberry silk was supplied by silkworm farmers (Suzhou, China). LiBr and polyethylene glycol (PEG; *M*_n_ = 12,000, Analytical Reagent) were purchased from Yuanye Bio-Technology Co. Ltd. (Shanghai, China). α-DMEM and penicillin/streptomycin were from Gibco, Thermo Fisher Scientific (USA). Fetal bovine serum was from Gibco (Australia). Live/dead staining kit was from Invitrogen (USA). Fluorescein isothiocyanate (FITC) phalloidin staining kit was from Sigma (USA). CD68 monoclonal antibody, CD206 monoclonal antibody, inducible nitric oxide synthase (iNOS) monoclonal antibody, and secondary antibody were from Abcam (UK). Sprague–Dawley (SD) rats were from Schlumberger Technology Co. Ltd. (Beijing, China).

### Construction of integrated SF scaffolds with biomimetic microchannel

Research has confirmed that the AF is composed of 15 to 25 loosely connected concentric rings of highly organized collagen fibers (lamellae). In every lamella, the collagen fibers lie parallel to each other and are orientated at approximately ±30° to the transverse axis. The primary goal of the scaffold we developed for this study was to achieve natural regeneration of IVD tissue by guiding tissue growth through microchannel structures. In this study, melt spinning technology was first used to prepare oriented cross PCL sacrificial templates (with oriented cross angle of 60°) for reconstruction of complex structure of the AF. Then, reverse pore-forming technology was used to prepare AF scaffolds with biomimetic microchannels. The central NP tissue was formed into a highly connected porous structure through paraffin sphere pore-forming technology. This technique of using microchannels for biomimetic reconstruction of IVD microstructures has not been reported in previous studies. The design of the BMI-SF scaffold structure took into account the fact that the in vivo repair effect of scaffold transplantation would be assessed at a later stage using rats weighing 300 to 350 g, and therefore, the scaffold dimensions, including the scaffold thickness of 1 mm, the thickness of the AF area of 1 mm, the diameter of the NP area of 2 mm, and the total scaffold diameter of 4 mm, were designed based on the caudal IVD of rats at this body weight.

#### Fabrication of oriented cross PCL sacrificial templates

Oriented cross PCL sacrificial templates were fabricated by melt spinning technology [36]. The dimensions of the PCL sacrificial template, including the 60° cross angle and diameter of PCL fibers, were designed to simulate the arrangement structure of natural collagen fibers. We chose PCL fibers with a diameter of mainly between 90 and 110 μm to reconstruct the collagen fiber arrangement structure. The AF scaffold with a circular microchannel structure was prepared after using reverse pore-forming technology. These oriented microchannels can guide cell-oriented growth and facilitate the orienting deposition of extracellular matrix, which is beneficial for the regeneration of tissues with oriented structures such as the AF. Briefly, PCL pellets were added into the melting reactor and melted into liquid at a temperature of 210 °C. The PCL was extruded through a 16-gauge needle and recovered by coiling it around a 2-mm-diameter revolving stainless steel mandrel. The melt spinning parameters were set as follows: collection distance of 1.5 cm, mandrel rotation speed of 100 rpm, horizontal table movement speed of 63 Hz, and solution extrusion rate of 1.5 ml/h. After removing the mandrel, a tubular sacrificial template with an inner diameter of 2 mm, an outer diameter of 4 mm, and PCL fibers arranged at a cross angle of 60° was obtained.

#### Preparation of paraffin spheres

Paraffin spheres were synthesized using lotion dispersion and chill-solidification techniques. Thirty grams of paraffin material was incorporated into 600 ml of a 0.5% polyvinyl alcohol (PVA) solution and heated in a constant temperature water bath at 80 °C until the paraffin was entirely melted. The mixture was stirred vigorously to create uniform paraffin spheres, followed by additional stirring and the rapid addition of ice to cool and solidify the paraffin spheres. The PVA was thoroughly washed off with distilled water and subsequently dried, and finally, the size of paraffin spheres is sifted with a gradient screen mesh. The prepared paraffin spheres were stored at 4 °C for subsequent experiments. In our previous study, we have found that the hierarchical macro-/microporous SF scaffolds possess controllable pore size, high porosity and interconnectivity, as well as certain mechanical property, which were able to promote cell attachment and proliferation [37]. Because the cells in the NP are scattered in the gel-like matrix, the hierarchical macro-/microscopic SF scaffolds are suitable for the regeneration of the NP tissue. In this study, 100- to 120-mesh paraffin spheres were selected to reconstruct the highly connected macropore structure in the NP area, where macropores (with pore size of 170 μm) are conducive to cell adhesion, proliferation, and extracellular matrix deposition, and interconnected pores are conducive to internal migration of cells.

#### Preparation of SF solution

The SF solution was formulated in accordance with established protocols [38,39]. First, the purchased mulberry silk was degummed by Na_2_CO_3_ solution with a concentration of 0.02 M. Second, we cut the degummed silk into pieces and dissolved it in 9.3 M LiBr solution. After the silk was completely dissolved, it was transferred to dialysis bag with molecular weight of 12,000 for dialysis. Then, the molecular weight of a dialysis bag of 3,500 was used to concentrate the SF solution in 20% PEG solution after the LiBr was completely removed. Finally, the concentration of SF solution was modified to 15% and preserved at 4 °C for future application.

### Morphology and size parameter characterization of pore-forming template

Oriented cross PCL fiber scaffold and paraffin spheres were observed by stereomicroscope (Leica, Germany) and scanning electron microscope (SEM; Phenom, Netherlands). A minimum of 50 fibers and their corresponding angles were selected at random from SEM pictures of 5 PCL fiber scaffolds, and then ImageJ software was utilized to quantify fiber diameter and angle measurements. At least 100 paraffin spheres were randomly selected from SEM images with a mesh size of 100 to 120 to measure their diameter.

### Preparation of PCL fiber patches

In order to prevent displacement of the BMI-SF scaffold and control scaffold in the IVD replacement model, the scaffold was fixed using PCL patches fabricated by electrostatic spinning technology with specific methods according to a previous study. The PCL patches were cut to the appropriate size (width 3 mm, length 7 mm) according to the size of caudal vertebrae (Fig. S1A).

### Characterizations of the BMI-SF scaffold

#### Morphology of scaffolds

The macroscopic morphology and dimensions of the BMI-SF scaffold were examined using a digital camera (Cannon EOS 1500D, Japan) (Fig. S1B). The cross-sectional and sagittal-sectional microstructure of the BMI-SF scaffold was characterized by stereomicroscope and SEM (accelerating voltage, 10 kV), and the diameter, thickness, and pore size of scaffolds were measured by ImageJ software. The BMI-SF scaffold and control scaffold were scanned with micro-CT (computed tomography) (Sky-Scan 1276, USA), and CTvox software (version 3.3.0.0) was used to reconstruct 3D images to demonstrate the difference of microchannel structure in different regions of the SF scaffold, including AF area and NP area.

#### Material composition characterization

The BMI-SF scaffold, control scaffold, PCL, and paraffin raw material were fixed on the stage of the Fourier transform infrared (FTIR)–Raman spectrometer (Bruker, Germany), and the spectrum of scaffolds between 500 and 3,500 cm^−1^ was recorded by the Raman spectrometer. For Raman spectroscopy detection, the scanning conditions were set as follows: laser power was 400 mV, scanning times was 200 times, and spatial resolution was 4 cm^−1^. Four groups of detection samples were scanned, and 5 replicate samples were randomly selected from each group.

#### Porosity of scaffolds

The porosity of SF scaffolds was quantified using the liquid displacement method [40,41]. The porosity was calculated based on the volume change before and after immersing the scaffold in the alcohol. The calculation formula is as follows:

$$\text{Porosity}=\left(V_{1}-V_{3}\right)/\left(V_{2}-V_{3}\right)$$ (1)

*V*_1_ denotes the volume of ethanol utilized to submerge the scaffold, *V*_2_ represents the total volume of ethanol and scaffold subsequent to the immersion, and *V*_3_ indicates the residual volume of ethanol following the extraction of the impregnated scaffold. Five samples from each group were selected for repeated measurements, and the average was calculated.

#### Swelling ratio of scaffolds

The swelling ratio of SF scaffolds was evaluated by the volume change before and after immersion in 1× phosphate-buffered saline (PBS) solution for 2 h. The swelling ratios were determined using the following equation:

$$\text{Swelling ratio}=\frac{V_{1}-V_{0}}{V_{0}}\times 100\%$$ (2)

*V*_0_ represents the volume of the dry scaffold, while *V*_1_ denotes the volume of the scaffold saturated with PBS. Five samples were identified for each category.

#### In vitro degradation

The degradation of SF scaffolds was carried out in 4 ml of PBS (pH 7.4). Briefly, SF scaffolds (BMI-SF scaffold and control scaffold) were cut into a cylinder (4 mm diameter and 4 mm height), weighted (*W*_0_), and then incubated in PBS at 37 °C for 1, 3, 5, 7, 10, 14, and 28 d. The scaffolds were taken out from PBS at a predetermined time point and washed with distilled water. The scaffolds were weighed again (*W*_1_) after they were dried. The degradation rates of SF scaffolds were calculated by the following equation:

$$\text{Weight loss}=\frac{W_{0}-W_{1}}{W_{0}}\times 100\%$$ (3)

where *W*_0_ is the dry weight of the scaffolds before degradation test and *W*_1_ is the volume of scaffold absorbed PBS. Five samples were detected for each group.

#### Mechanical properties

The mechanical compressive properties of the scaffold were evaluated using a mechanical testing apparatus (Instron 5865, USA). To assess compressive capabilities, the scaffolds were immersed in PBS for 2 h and subsequently sectioned into cylinders with a height of 8 mm. The instrument parameters were established as follows: compression speed of 0.2 mm/s and compression height of 4 mm (strain rate range from 0% to 50%). Data were collected with Merlin v5.31 software, and the stress–strain curves were generated with GraphPad Prism 8 (GraphPad Software, La Jolla, CA, USA). The compression modulus and the tensile modulus were determined. Each group underwent testing with a minimum of 5 repeated samples.

#### In vitro biocompatibility evaluation

Rat bone marrow mesenchymal stem cells (BMSCs) were extracted from the tibia and femur of SD rats following the previously outlined protocol [36]. The cells were cultured in α-DMEM supplemented with 10% fetal bovine serum and 1% penicillin/streptomycin under humidified conditions with 5% CO_2_ at 37 °C. Subsequently, purified cells were utilized in the experiments.

Before cell culture, the scaffolds were cut to 1 mm thickness and sterilized with the radioisotope cobalt 60 (Co60) at a dose of 15 Gy. The scaffolds were soaked in sterile 1× PBS solution for 12 h to fully hydrate, and then 3T3 cells were seeded onto the scaffolds for continuous culture. Live/dead staining and the Cell Counting Kit-8 (CCK-8) method were used to evaluate cell viability and proliferation within the scaffold. BMSCs were seeded onto the scaffolds and cultured for 3 d. Furthermore, cells were labeled with FITC–phalloidin to examine the morphology of cells within the scaffold and count the percentage of oriented cells in the AF area and NP area. Following a 3-d culture period, the cell–scaffold complex was fixed using glutaraldehyde and subsequently lyophilized under vacuum; the morphology of the cells on the scaffold surface was then examined via SEM.

#### Cell infiltration and biocompatibility in vivo

The BMI-SF scaffold and control scaffold were implanted subcutaneously on the back of SD rats. All rats were sacrificed 2 and 4 weeks after operation, and the scaffolds were collected. The morphology of scaffolds and the surrounding tissue envelope were observed using a stereomicroscope. Cell infiltration and vascular ingrowth were assessed using hematoxylin and eosin (H&E) staining. Immunofluorescence staining of macrophage markers CD68, iNOS, and CD206 was used to analyze the inflammatory response at 2 and 4 weeks after implantation.

### Animal experiment

#### Animal model and scaffold implantation

The BMI-SF scaffold was implanted into the caudal spine 7/8 of SD rats to evaluate its function. All experimental animals received approval from the Animal Ethics and Welfare Committee, and the procedures adhered to the rules set forth by the Institutional Animal Care and Use Committee of Tianjin Hospital (no. 2020-YLS-012). Thirty male SD rats, weighing between 300 and 350 g, were recruited to establish an animal model for mechanical IVD replacement. The rats were sedated with pentobarbital sodium (40 mg/kg, Sigma) and secured in a prone position. The vertebral column was exposed, and native discs were removed to prepare the space for insertion of the scaffold into the tail. After discectomy, the control scaffold was implanted into the caudal spine 5/6 and the biomimetic microchannel SF scaffold was implanted into the caudal spine 7/8. The caudal spine 8/9 was defined as the discectomy group in which only the IVD was removed. To prevent the implanted scaffolds from leaving the spinal space after implantation, we fixed the IVD segment implanted with the scaffold using homemade PCL patches prepared by electrospinning (Fig. S1A). The wound was sutured after washing with normal saline containing gentamicin.

#### Magnetic resonance imaging evaluation

To assess disc height and IVD recovery, a magnetic resonance imaging (MRI) (Philips Ingenia 3.0T, Holland) of the caudal spine was obtained after anesthesia. A high-resolution T2-weighted sequence (resolution: 104.2 μm × 78.1 μm × 1 mm) was acquired at 2, 4, and 12 weeks post-operation. The disc height index (DHI) quantifies the height of the IVD relative to the length of the vertebral body. The height of the disc space and the length of the vertebral body in each surgical segment of the MRI image are quantified using ImageJ software to compute the DHI. The formula for calculation is as follows:

$$\mathrm{DHI}=\frac{2H_{D}}{L_{\mathrm{VB}1}+L_{\mathrm{VB}2}}$$ (4)

where *H*_D_ is the height of the disc space and *L*_VB1_ and *L*_VB2_ are the length of 2 adjacent vertebral bodies of the IVD.

#### Biomechanical tests

The scaffold implanted motion segments and intact native motion segments were acquired by excising the surrounding tissue at 4 and 12 weeks post-operation. The compressive modulus of the motion segment was evaluated using a dynamic mechanical analysis apparatus (Instron 5865, USA) at a compression rate of 0.01 mm/s. The apparent modulus was determined as the slope of the linear segment of the stress–strain curve.

#### Histological evaluation

For histological analysis, the removed tail segments were fixed in 4% paraformaldehyde for at least 3 d, sequentially decalcified in 10% EDTA solution (pH 7.0, Solarbio) at 37 °C for 6 to 8 weeks, then embedded in paraffin, and sectioned at 5-μm thickness. Sections from each group were stained with H&E, Picrosirius red, and Alcian blue to observe how the replacements integrate with adjacent vertebrae and tissue components in disc space.

#### Statistical analysis

All quantitative data for independent parallel experiments were presented as mean ± SD. SPSS software and GraphPad Prism software were used for statistical analysis. Statistical analysis of data involved one-way analysis of variance (ANOVA) and Tukey’s post hoc analysis. Differences between groups were considered statistically significant at a value of *P < 0.05, ***P < 0.001, ns: no significant.

## Results

### Fabrication of the BMI-SF scaffold

The prepared PCL sacrificial templates were cut to a length of 1 cm and placed into silicone tubes with an inner diameter of 4 mm. Then, the paraffin spheres (size, 100 to 120 mesh) were filled into the center of the PCL sacrificial templates and preheated at 55 °C for 20 min to make the paraffin spheres partially adhere. The assembled complete sacrificial molds were perfused with 15% SF solution, and the interior of the sacrificial molds was completely perfused with SF solution by repeated vacuum suction with a vacuum pump. Then, they were transferred to liquid nitrogen for freeze-forming, and finally, the freeze-formed composite scaffolds were transferred to a low-temperature vacuum dryer for continuous freeze-drying. The SF/PCL/paraffin composite scaffolds were obtained by removing the excess SF material and the outer silicone tube and then immersed in absolute ethanol for 2 h to convert the SF from the α-helical structure to the β-sheet structure. Finally, PCL and paraffin in the composite scaffolds were leached out by dichloromethane and hexane, and the BMI-SF scaffold was successfully fabricated. The BMI-SF scaffold prepared above was cut into 1-mm-thick cross sections for subsequent experiments. The scaffolds in the control group were SF scaffold without microchannels fabricated by freeze-drying technology. The complete process of the BMI-SF scaffold preparation is shown in Fig. 1.

### Characterization of pore-forming template

**Fig. 2.**
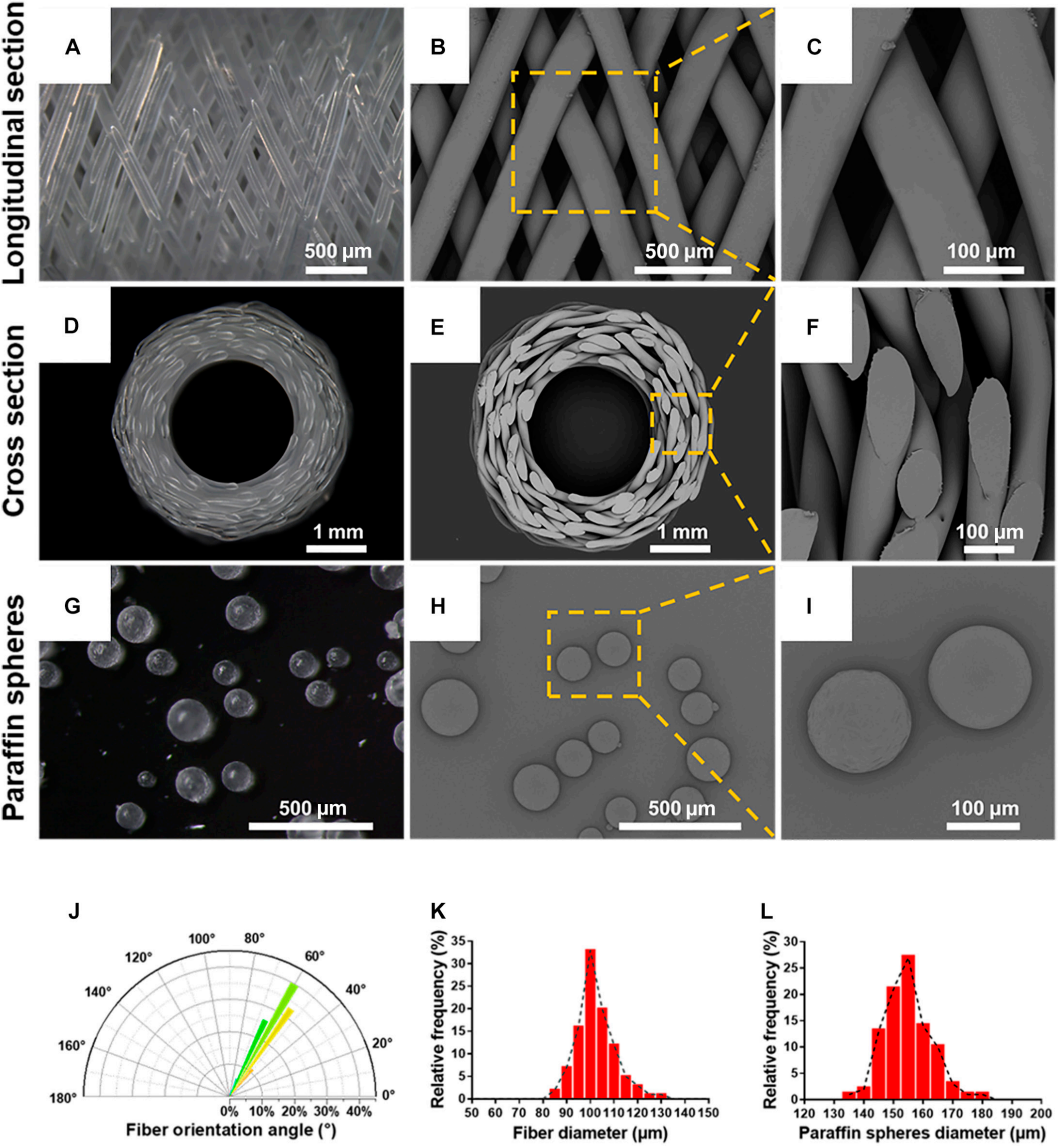
Characterization of the pore-forming template. (A) Stereomicroscopic image of the PCL fiber scaffold in longitudinal section. (B and C) SEM images showing the surface morphology of the PCL fiber scaffold. (D) Stereomicroscopic observation of the PCL fiber scaffold in cross section. (E and F) SEM images showing the surface morphology of the PCL fiber scaffold. (G) Stereomicroscopic image showing the gross appearance of paraffin spheres. (H and I) SEM images showing the surface morphology of paraffin spheres. (J) PCL fiber cross angle measured by stereomicroscopic images. (K and L) Diameters of PCL fibers and paraffin spheres.

We used stereomicroscope and SEM to observe the surface structure of the PCL fiber scaffold. The longitudinal section showed that the fibers were uniformly thick and cross-layered (Fig. 2A to C), and the average cross angle of the PCL fiber was 59.2 ± 5.89° (Fig. 2J). The diameter of PCL fiber is mainly between 90 and 110 μm (Fig. 2K). The cross section showed that the PCL fiber scaffold had a circumferentially oriented multilayer structure, and high-magnification images showed partial adhesion at the interface of the PCL fiber (Fig. 2D to F). The paraffin spheres were regularly rounded, SEM images showed that they had a smooth surface (Fig. 2G to I), and the average diameter of the paraffin spheres was 152.9 ± 10.23 μm (Fig. 2L).

### Characterization of SF scaffold

#### Morphology

**Fig. 3.**
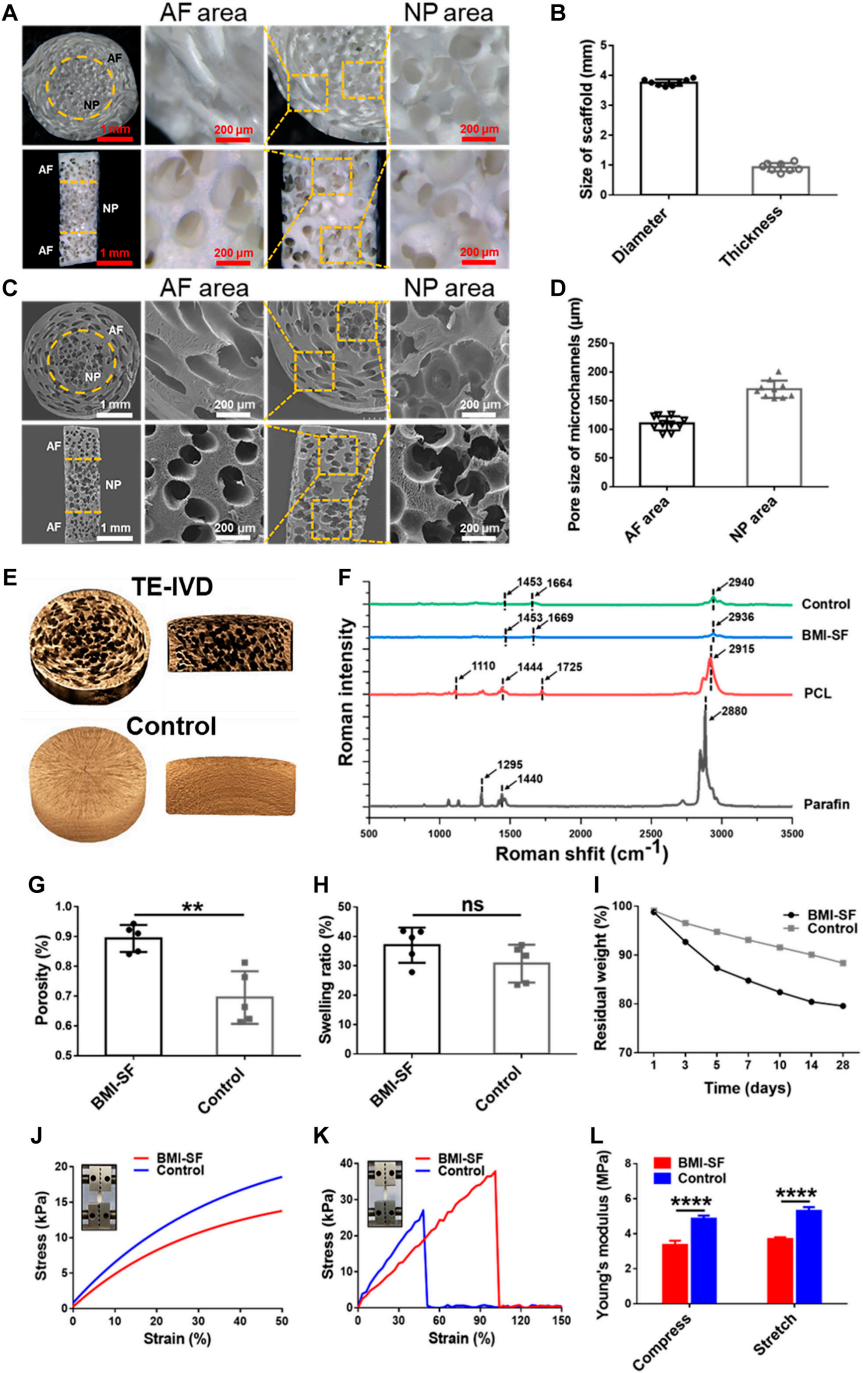
Characterization of the structural and physicochemical properties of the BMI-SF scaffold. (A) Stereomicroscopic images of the BMI-SF scaffold in cross section and longitudinal section. (B) Diameter and thickness measurement of the BMI-SF scaffold. (C) SEM images showing the microchannel structure of the BMI-SF scaffold in cross section and longitudinal section. (D) Microchannel pore size in the AF area and NP area of the BMI-SF scaffold. (E) 3D micro-CT reconstruction images showing the internal structure of the BMI-SF scaffold compared to the control scaffold. (F) Raman spectroscopic detection of material composition in different scaffolds. (G) Porosity of the BMI-SF scaffold and the control scaffold. (H) Calculation of the swelling rate of the BMI-SF scaffold and the control scaffold after soaking in PBS for 2 h. (I) Degradation curve of the BMI-SF scaffold and the control scaffold. (J) Stress–strain curves for compression testing of the scaffold. (K) Stress–strain curves for tensile testing of the scaffold. (L) Calculation of the compression modulus and tensile modulus of the BMI-SF scaffold and the control scaffold. Bar heights and error bars represent means ± SD. Statistical analysis is presented as follows: **P < 0.01, ****P < 0.0001, ns: no significant.

The BMI-SF scaffold has a disc-shape appearance with a diameter of 4 mm and a thickness of 1 mm (Fig. 3A and B). The microstructure of the BMI-SF scaffold was visualized by SEM (Fig. 3C), showing the structural differences between the AF area and the NP area of the scaffold. The cross-sectional images showed that the AF area was located at the periphery of the scaffold and had a concentrically arranged intersecting microchannel structure and cross-connection between some of the microchannels, while the NP area was located in the center of the scaffold and had a highly interconnected porous structure. Longitudinal sectional images showed that the AF area was located at the upper and lower ends of the scaffold, and the NP area was located in the middle of the scaffold. Microchannels in the AF area have a uniform inner diameter, are regularly arranged, and are connected to each other at intersections. The inner diameter of microchannels in the AF area was 110 ± 15 μm. The NP area exhibits a highly interconnected irregular porous structure with a measured pore size of 170 ± 22 μm (Fig. 3D). 3D micro-CT images showed that the BMI-SF scaffold has a highly interconnected microchannel structure in the AF area and an irregular porous structure in the NP area. Meanwhile, without spatial separation, the 2 parts of the AF area and NP area were only structurally different and well fused into an integrated scaffold. In contrast, the control scaffold was a solid cylinder without microchannels and porous structure due to the lack of sacrificial templates for pore formation. There was no obvious structural difference between the peripheral and central areas (Fig. 3E). The 3D micro-CT reconstruction animation demonstrated the BMI-SF scaffold with different microchannel structure in the AF area and NP area more visually (Movies S1 and S2).

#### Chemical composition

FTIR–Raman spectroscopy analysis confirmed that the chemical composition of the BMI-SF scaffold was pure SF. We observe characteristic peaks of the BMI-SF scaffold at 1,453, 1,669, and 2,936 cm^−1^, which were consistent compared to the control scaffold (pure SF composition) and consisted of 3 characteristic peaks at 1,453, 1,664, and 2,940 cm^−1^. Furthermore, the characteristic PCL peaks at 1,725 and 2,915 cm^−1^ as well as characteristic paraffin peaks at 1,295 and 2,880 cm^−1^ were not found in the BMI-SF scaffold (Fig. 3F).

#### Porosity and mechanical property

We evaluated the porosity of the BMI-SF scaffold and the control scaffold using the liquid displacement method. The findings indicated that the porosity of the BMI-SF scaffold (89.33 ± 6.58%) was markedly greater than that of the control scaffold (69.52 ± 5.25%) (Fig. 3G). After soaking in PBS for 2 h, the swelling experiment results showed that the swelling rate of BMI-SF scaffold was slightly higher than that of the control scaffold; nevertheless, no statistical significance was seen between the 2 groups (Fig. 3H). The degradation results indicated that the BMI-SF scaffold exhibited a more rapid disintegration rate compared to the control scaffold (Fig. 3I). To assess the mechanical qualities of scaffolds, they were sectioned into cylinders of 8 cm in height. Strain–stress tests indicated that the stress of the BMI-SF scaffold escalated gradually with increased stretch, whereas the stress of the control scaffold exhibited a rapid escalation with increased stretch, implying that the microchannel architecture enhances the porosity of the SF scaffold while diminishing its mechanical strength (Fig. 3J and K). The Young’s modulus measurements for compression and tension indicated that the BMI-SF scaffold exhibited markedly lower values than the control scaffold, implying the necessity to consider the degradation of mechanical characteristics of the scaffold during the construction of microchannel structures (Fig. 3L).

### Cell behavior in scaffolds

**Fig. 4.**
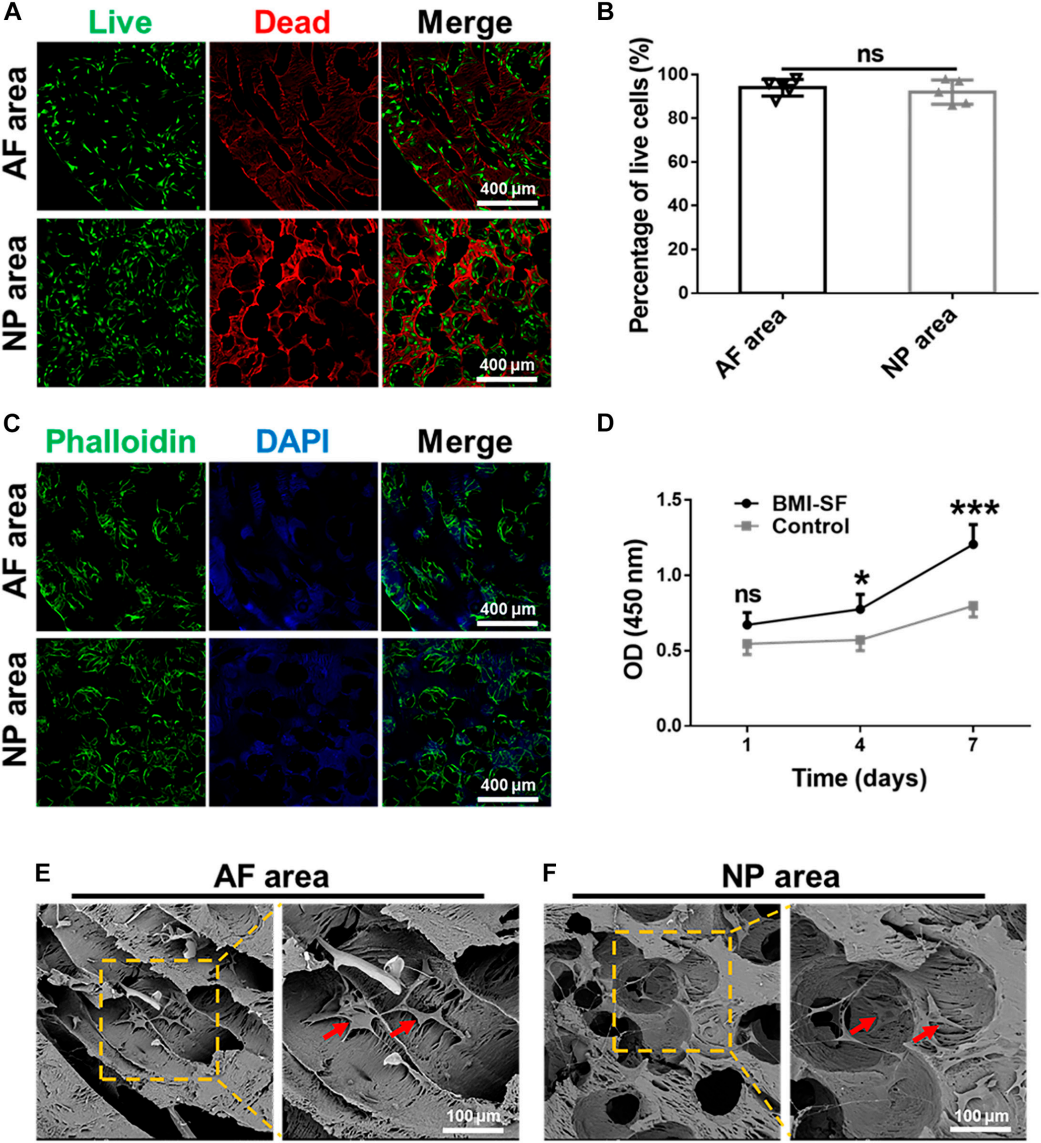
Characterization of cell viability and morphology on the BMI-SF scaffold. (A and B) Live/dead staining of cells seeded on the BMI-SF scaffold at day 3. Live cells stained with green fluorescence and dead cells stained with red fluorescence (nonspecific red staining of SF shows the microchannel structure in different areas of the BMI-SF scaffold). (C) FITC–phalloidin and DAPI staining of cells seeded on the BMI-SF scaffold showing cell morphology and cell infiltration in microchannels (cytoskeleton stained green and cell nuclei stained blue; DAPI nonspecific SF staining shows microchannels in different areas of the BMI-SF scaffold). (D) Cell proliferation on the BMI-SF scaffold and control scaffold was determined by CCK-8 assay at days 1, 3, and 5. (E and F) SEM images showing the cell morphology and distribution on the BMI-SF scaffold. Red arrows indicate cells attached to the surface of microchannels. Statistical analysis is presented as follows: *P < 0.05, ***P < 0.001, ns: no significant.

An ideal tissue engineering scaffold should have good biocompatibility and cell infiltration ability. Cell viability assay was performed by live/dead staining after inoculation of 3T3 cells on the BMI-SF scaffold for 3 d. The results showed that cells had good adhesion and viability on the surface of the BMI-SF scaffold: Green fluorescent indicated living cells growing adherently on the surface of microchannels in the AF area and the inner wall of the pore in the NP area (Fig. 4A). The cell counting results showed that the percentage of live cells was 94 ± 1.7% and 92 ± 2.5% in the AF area and NP area, respectively, with no marked difference between them, suggesting that cells can easily adhere to the surface of microchannels in both the AF area and NP area and survive well (Fig. 4B). By FITC–phalloidin staining, the cytoskeleton can exhibit green fluorescence, and the spreading morphology of green fluorescence can indirectly reflect whether cell growth is oriented or not. After 3 d of cell seeding on the BMI-SF scaffold, FITC–phalloidin staining showed that a large number of cells with green fluorescence were attached to the surface of the scaffold. Cells showed different cytoskeletal morphology in different microchannels of the BMI-SF scaffold. The BMI-SF scaffold was easily stained by 4′,6-diamidino-2-phenylindole (DAPI) and showed nonspecific blue fluorescence, which helps us distinguish between the AF area and NP area. A large number of green fluorescent cells can be seen adhering and growing on the inner wall surface of microchannels in the AF area. High-magnification images showed that cells grow in a long spindle shape along the inner wall of microchannels, and the cytoskeleton has an orientation. Many green fluorescent cells can be seen on the inner surface of the pore in the NP area. High-magnification images showed that cells adhere to the inner wall of the pore and grow without orientation (Fig. 4C). The cell counting results showed that the percentage of oriented cells was 87 ± 2.2% and 15 ± 4.5% in the AF area and NP area, respectively (Fig. S2). The result of CCK-8 showed that the optical density (OD) value of both the BMI-SF scaffold and the control scaffold increased from day 1 to day 7, indicating that cells on both scaffolds have good proliferation ability. The OD value of the BMI-SF scaffold was higher than that of the control scaffold. Although there was no marked difference in OD value between the 2 groups on the first day (*P* > 0.05), the difference in OD value between the 2 groups cultured for 4 and 7 d was significant (*P* < 0.05), which may be mainly due to microchannels of the BMI-SF scaffold providing more sufficient growth space for cells and facilitating cell proliferation (Fig. 4D). Cell morphology was observed on the surface of different microchannels using SEM after culturing the cell–scaffold complex for 3 d. The results showed that cells on the microchannels in the AF area had orientation, while cells on the surface of the NP area had no orientation. Red arrows indicate cells attached to the surface of microchannels (Fig. 4E and F).

### In vivo biocompatibility assessment of scaffolds (subcutaneous implantation experiments)

**Fig. 5.**
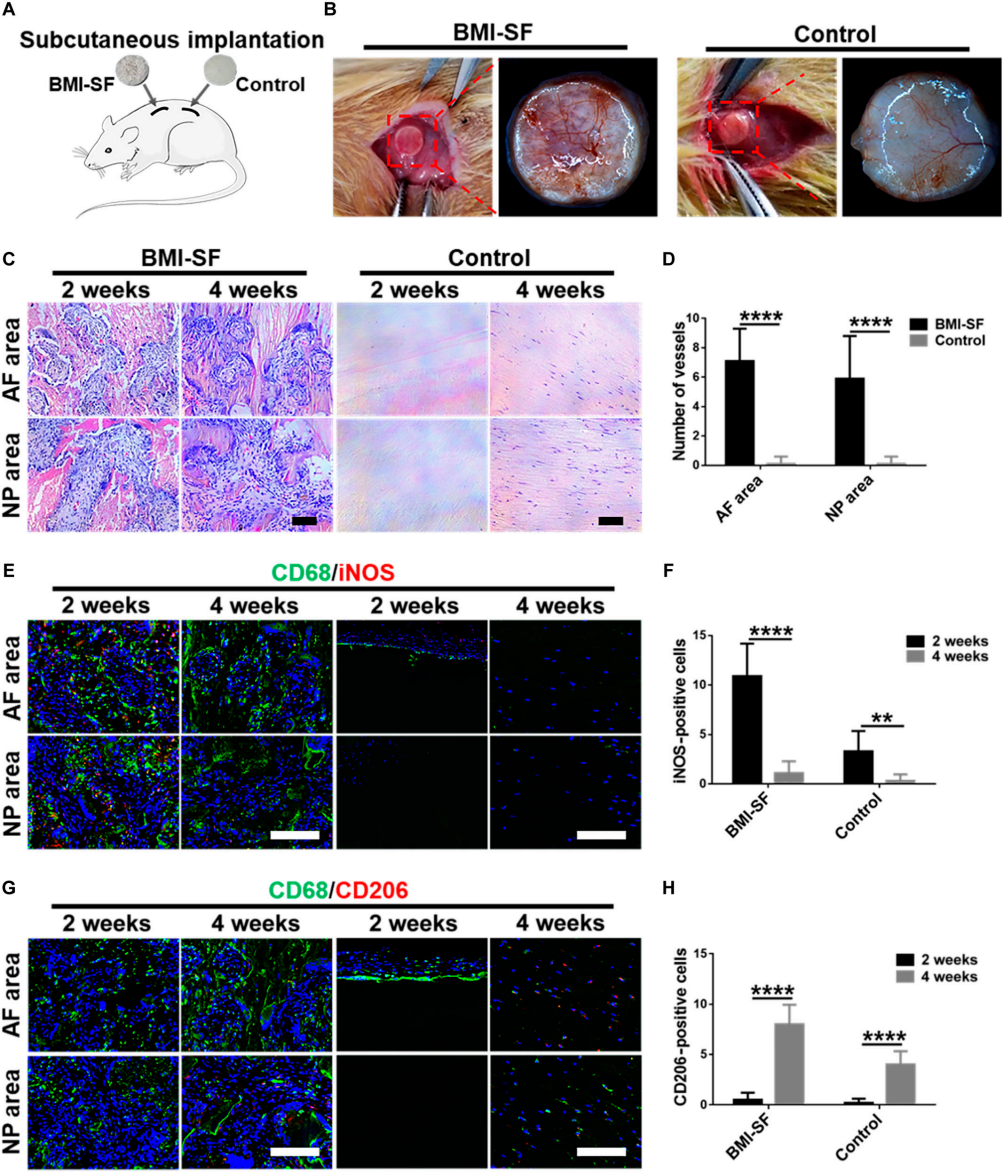
Cell infiltration, vascularization, and immunogenicity of the BMI-SF scaffold and control scaffold in the rat subcutaneous implantation model after 2 and 4 weeks. (A) Schematic illustrating the establishment of subcutaneous implantation model in rats. (B) Representative photos of the BMI-SF scaffold and control scaffold after 4 weeks of implantation. (C) H&E staining showing cell infiltration and neovessels in the BMI-SF scaffold through microchannels at 2 and 4 weeks compared to the control scaffold. (D) Quantification of neovessels (*n* = 15). (E) Macrophage polarization was detected by double staining for iNOS (M1, pro-inflammatory, red)/CD68 (M0, pan-macrophages, green). (F) Quantification of iNOS-positive cells (*n* = 15). (G) Macrophage polarization was detected by double staining for CD206 (M2, anti-inflammatory, red)/CD68 (M0, pan-macrophages, green). (H) Quantification of CD206-positive cells (*n* = 15). Statistical analysis is shown as **P < 0.01, ****P < 0.0001. Scale bars, 100 μm (C) and 200 μm (E and G).

Subcutaneous implantation experiments were used to evaluate the in vivo biocompatibility of the BMI-SF scaffold and their ability to guide tissue regeneration (Fig. 5A). While sampling the subcutaneous implanted scaffolds for 2 and 4 weeks, it was found that both the BMI-SF scaffold and the control scaffold integrated well with the subcutaneous tissue of rats. The subcutaneous tissue wrapped around the scaffold was firmly attached to the scaffold and was difficult to separate. No obvious inflammatory reaction was observed around the BMI-SF scaffold and the control scaffold, suggesting that the SF scaffold has good in vivo biocompatibility and can be used as a graft for tissue regeneration (Fig. 5B). Observation under the stereomicroscope revealed the presence of a transparent tissue wrapping around both the BMI-SF scaffold and control scaffold, and the newborn blood vessels within the tissue wrapping could be clearly observed. Furthermore, the porous structure of the BMI-SF scaffold can be observed through the tissue wrapping, with blood vessels extending deep into the scaffold along microchannels. In contrast, the control scaffold showed no porous structure and blood vessels only grew on the surface of the scaffold. H&E staining of the sagittal section showed that microchannels of the BMI-SF scaffold were infiltrated with a large amount of tissue and cells, and many new blood vessels were visible. When the number of new blood vessels was counted in the scaffold implanted subcutaneously for 4 weeks, it was found that there were almost no new blood vessels in the control scaffold. The number of new blood vessels in the AF area of the BMI-SF scaffold was 7.1 ± 2.2 and in the NP area was 5.9 ± 2.9, both of which were markedly higher than those in the control scaffold (*P* < 0.0001) (Fig. 5C and D). Immunofluorescence staining was used to evaluate the infiltration and polarization of macrophages in different scaffolds, and the numbers of pan-macrophages (M0), pro-inflammatory macrophages (M1), and anti-inflammatory macrophages (M2) were identified by immunofluorescence staining of CD68, iNOS, and CD206, respectively. Double staining for CD68/iNOS showed that after 2 weeks there were more M1 macrophages with iNOS-positive expression around and within the BMI-SF scaffold, while the number of M1 macrophages decreased markedly after 4 weeks of subcutaneous implantation (*P* < 0.0001). The number of M1 macrophages with iNOS-positive expression around the control scaffold decreased markedly from 2 to 4 weeks (*P* < 0.01) (Fig. 5E and F). On the contrary, double staining for CD68/CD206 showed that after 2 weeks, only a small number of CD206-positive M2 macrophages were located within the BMI-SF scaffold or around the control scaffold. However, the number of M2 macrophages increased markedly after 4 weeks of subcutaneous implantation compared to the scaffolds after 2 weeks (*P* < 0.001) (Fig. 5G and H).

To observe the tissue reconstruction guided by the BMI-SF scaffold in vivo, we performed cross-sectional sections of the subcutaneously implanted scaffolds. H&E staining showed that the BMI-SF scaffold remained intact as a disc-shaped scaffold with tissue infiltration within the BMI-SF scaffold at 2 and 4 weeks after implantation. Subcutaneous implantation for 2 weeks mainly fills the microchannels with tissue, while subcutaneous implantation for 4 weeks results in greater infiltration of tissue in microchannels of the BMI-SF scaffold. More new blood vessels can be seen in microchannels, and more cells begin to migrate into the wall of the BMI-SF scaffold, which may be related to the degradation of the BMI-SF scaffold in vivo (Fig. S3).

### MRI of rat caudal spine

**Fig. 6.**
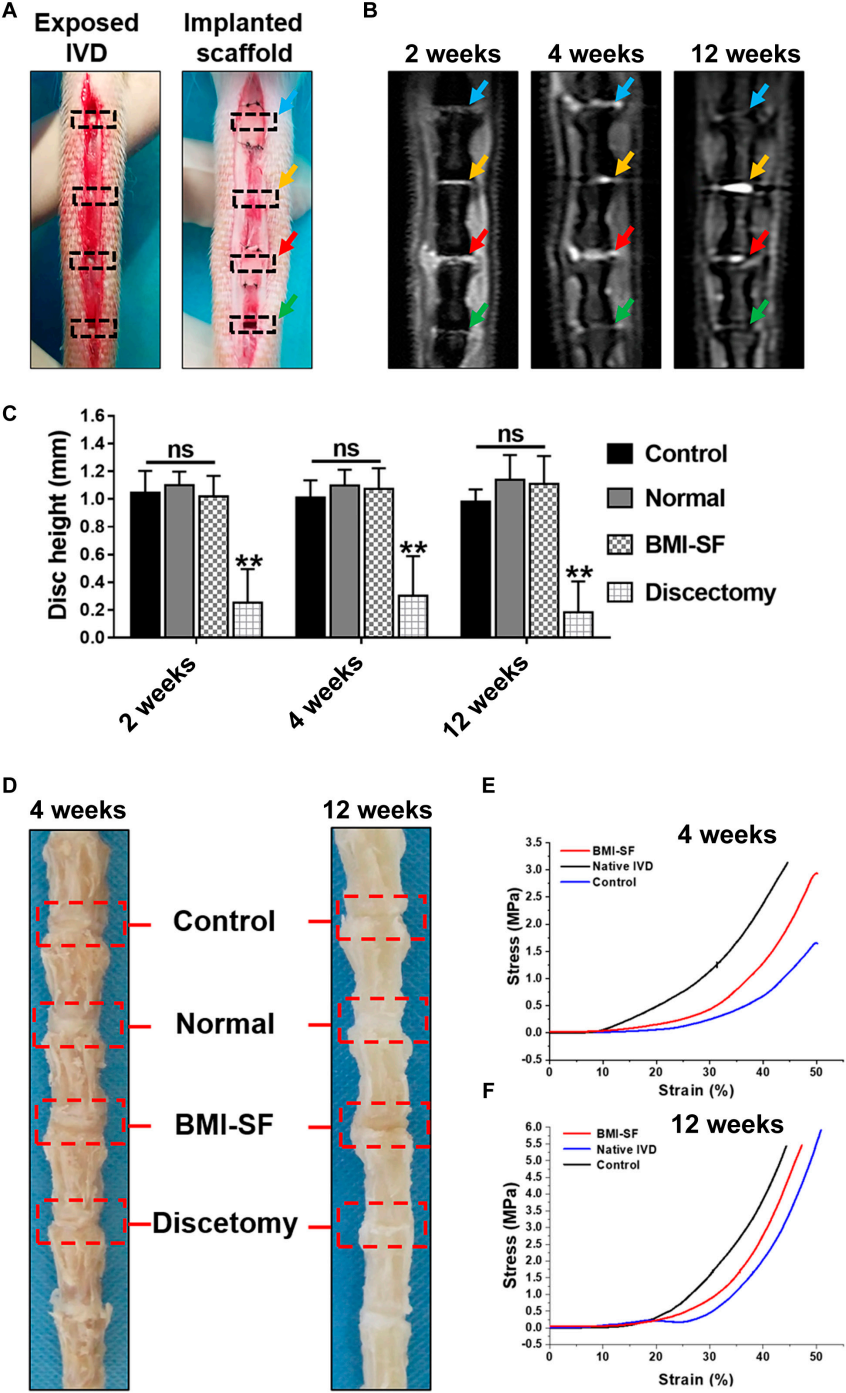
In vivo assessment of the BMI-SF scaffold and the control scaffold in the rat caudal spine. (A) Intraoperative images showing the exposed IVD and sutured patch after scaffold implantation. The black box indicates the location of the disc space. Control scaffold implantation, normal IVD, BMI-SF scaffold implantation, and discectomy were marked by blue, yellow, red, and green arrows. (B) T2-weighted MRI of control scaffold implantation, normal IVD, BMI-SF scaffold implantation, and discectomy (marked by blue, yellow, red, and green arrows). (C) IVD height of different groups. (D) Dissection of the caudal spine 4 and 12 weeks after scaffold implantation. (E and F) Uniaxial compressive stress–strain curves for control scaffold and BMI-SF scaffold implanted motion segments. Statistical analysis is shown as **P < 0.01, ns: no statistical significance.

We divided the caudal spine into 4 groups: Caudal 2/3 disc space (Ca2/3) and caudal 4/5 disc space (Ca4/5) were implanted with control scaffold and BMI-SF scaffold, respectively. Caudal 3/4 disc space (Ca3/4) was a normal IVD set up as the normal control group, while caudal 5/6 disc space (Ca5/6) just underwent simple discectomy (Fig. 6A). T2-weighted MRI showed hydrated tissue in the disc space of Ca2/3 and Ca4/5 resembling normal IVD (Ca3/4) at 2 and 4 weeks postoperatively. However, Ca5/6 showed a black signal and marked loss of IVD height compared to the other 3 groups. T2-weighted MRI post-operation at 12 weeks showed that the signal intensity of Ca2/3 and Ca4/5 decreased compared to the same level at 2 and 4 weeks. Ca2/3 even appeared as a black signal, but the IVD height could still be maintained, and bright high signals were still visible in the Ca4/5 level. By measuring the IVD height, it was found that the height of Ca2/3 and Ca4/5 levels could be maintained at 2, 4, and 12 weeks after surgery and there was no marked decline over time. However, the IVD height of Ca5/6 was lost and the disc space was markedly narrower than that of Ca3/4 (*P* < 0.01) (Fig. 6B and C).

The dissection of caudal spine at 4 and 12 weeks after scaffold implantation showed that the control scaffold and the BMI-SF scaffold can integrate well with the adjacent vertebrae and create functional motion segments. The IVD height of the control scaffold and the BMI-SF scaffold implantation groups was maintained by soft tissue filling in the disc space, while adjacent vertebrae in the discectomy group fused together (Fig. 6D). The mechanical properties of the control scaffold and the BMI-SF scaffold were tested after 4 and 12 weeks of implantation. Both the BMI-SF scaffold and the control scaffold maintained the motion function after implantation, and the comparison revealed that the mechanical properties of the segments implanted with the BMI-SF scaffold were closer to those of the normal IVD motion segments, indicating that the BMI-SF scaffold is more suitable as an artificial graft for IVD replacement (Fig. 6E and F).

### Histological analysis

**Fig. 7.**
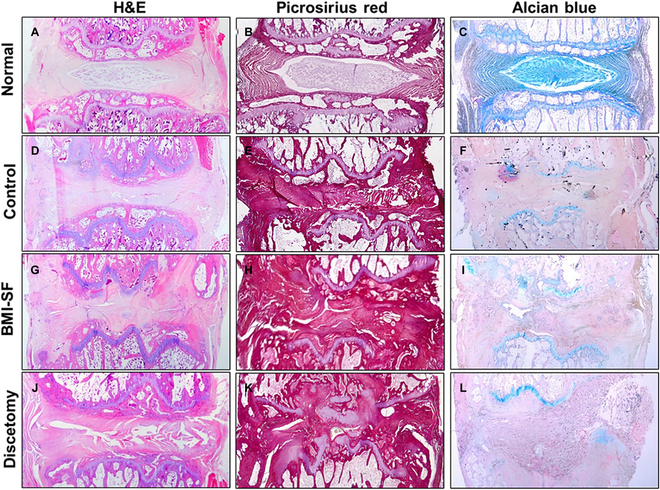
The histological analysis at 12 weeks after scaffold implantation. (A, D, G, and J) H&E staining of the native IVD, control scaffold, BMI-SF scaffold, and discectomy groups, respectively. (B, E, H, and K) Picrosirius red staining of the 4 groups. (C, F, I, and L) Alcian blue staining.

To determine whether the BMI-SF scaffold could integrate well with the adjacent vertebrae and guide tissue regeneration in situ, we analyzed the disc space 12 weeks postoperatively using H&E, Picrosirius red, and Alcian blue staining. In the native IVD group, the IVDs were structurally intact with an oval-shaped NP in the center surrounded by multilayered AF (Fig. 7A). The NP area was weakly stained with Picrosirius red and strongly stained with Alcian blue, indicating high glycosaminoglycan (GAG) content and low collagen content. In contrast, the AF area was strongly stained with Picrosirius red and weakly with Alcian blue, indicating high collagen content (Fig. 7B and C). In the discectomy group, the lack of soft tissue in the disc space resulted in loss of IVD height, and spinal fusion occurred due to the damage of cartilaginous endplate caused by the lack of soft tissue to absorb mechanical loads (Fig. 7J to L). In the control group with scaffold implantation, many soft tissues were visible in the disc space, and Picrosirius red and Alcian blue staining showed that the neo tissues were mainly collagen (Fig. 7D to F). In the BMI-SF scaffold implantation group, more soft tissues were visible and the IVD height was markedly restored compared to the discectomy group (Fig. 7G). Picrosirius red and Alcian blue staining showed that the neo tissues were collagen and a small amount of GAG (Fig. 7H and I).

## Discussion

Research has shown that tissue-engineered total IVD comprising both NP and AF has achieved great promise in the treatment of IVDD [40,41]. Yet, in these studies, NP and AF were usually constructed separately and assembled into a complete artificial IVD. This simple assembly process ignored the integrity of the NP and AF in the IVD. Tissue function is determined by the hierarchical structural organization of the tissue. Therefore, it is crucial to achieve biomimetic reconstruction of complex tissues such as IVD. In this work, we selected SF as scaffold material considering the biocompatibility, biodegradability, and mechanical properties of the materials. At the same time, we focused on the integrated reconstruction of the hierarchical structure of NP and AF. Therefore, we used reverse pore-formation technology to fabricate the BMI-SF scaffold and hypothesized that different microchannels in the NP and AF area can control the remodeling of different tissues and ultimately achieve IVD regeneration.

To develop artificial total IVD with microchannels, our strategy to fabricate the BMI-SF scaffold is as follows: (a) using melt spinning technology and phase separation technology to fabricate AF and NP pore-formation templates, (b) assembling AF and NP pore-formation templates to form a complete sacrificial template, and (c) combining freeze-drying with template elution technology to successfully obtain disc-shaped BMI-SF scaffold resembling natural IVD (Fig. 2 and Fig. S1). SEM and micro-CT confirmed that our prepared BMI-SF scaffold had different microchannels of NP and AF (Fig. 3C and E), and to further observe the internal microchannels of BMI-SF scaffold, we demonstrated this by a 3D reconstruction animation of micro-CT, which showed that the control scaffold had no microchannels, while the BMI-SF scaffold used microchannels to reconstruct the NP and AF structure, and the structure between the 2 parts was characterized as integrated (Movies S1 and S2). The IVD is an organ that absorbs mechanical loads to provide flexibility to the spine. Therefore, the mechanical properties of the biomimetic IVD scaffold will play a key role in tissue regeneration. We found that the BMI-SF scaffold could withstand the external compressive and tensile forces within a certain range (Fig. 3J to L), and it is worth noting that the microchannels reduce the mechanical properties of the BMI-SF scaffold, suggesting that we should prevent the excessive loss of mechanical properties when using microchannels for tissue reconstruction, which may lead to the collapse of microchannels under external loading.

The main application purpose of biomimetic IVD scaffold as artificial graft is to implant them into animals or humans. Therefore, it is necessary to test the biocompatibility of the scaffold before conducting in vivo studies. Live/dead staining of cells inoculated with BMI-SF scaffold showed that the cells had good adhesion and survival ability on the surface of microchannels in the NP and AF areas of the scaffold (Fig. 4A and B). In addition, the CCK-8 results showed that compared to the control scaffold, the proliferation ability of cells cultured on the BMI-SF scaffold for 3 and 5 d was markedly higher (Fig. 4A and D), which may be due to microchannels of the BMI-SF scaffold providing more growth space for cell proliferation. With increasing cultivation time, it can be observed that cells can migrate along microchannels into the interior of the BMI-SF scaffold. FITC–phalloidin staining showed that cells have good skeletal morphology on the surface of microchannels and that cells in the AF area grow in an oriented manner, while cells in the NP area have no orientation (Fig. 4C). The cell counting results showed that the percentage of oriented cells was 87 ± 2.2% and 15± 4.5% in the AF area and NP area, respectively (Fig. S2). Images of SEM also confirm that the cells are aligned in the AF area of the BMI-SF scaffold but not in the NP area (Fig. 4E and F). Surprisingly, these results demonstrated that we could effectively regulate cell growth behavior through different microchannels of the scaffold, which is crucial for reconstructing structurally complex IVD tissue. Prior to the IVD replacement model with the BMI-SF scaffold, we further investigated the biocompatibility of the BMI-SF scaffold and its regulatory effect on tissue regeneration by subcutaneous implantation (Fig. 5A). The BMI-SF scaffold was obtained after subcutaneous implantation for a period of time, and it was observed that the BMI-SF scaffold and the control scaffold were well integrated into the subcutaneous tissue without causing obvious graft rejection (Fig. 5B), which further proved that the BMI-SF scaffold has good biocompatibility and can be used as a graft for IVD replacement. H&E staining of cross-sectional sections of the scaffold showed that after 2 and 4 weeks of subcutaneous transplantation, the BMI-SF scaffold still retained its intact disc-shape appearance, and a large number of cells and tissues could be seen filling microchannels in the NP and AF areas (Fig. S2). H&E staining of the longitudinal section showed that it was difficult to infiltrate tissue into the control scaffold, while microchannels of the BMI-SF scaffold facilitated the migration of tissue into the scaffold and greater neovascularization was observed in microchannels, providing sufficient nutritional support for tissue remodeling and regeneration (Fig. 5C and D). Immunofluorescence results on macrophages showed that M1 macrophages were more and M2 macrophages less infiltrated in the local area after 2 weeks of BMI-SF scaffold and control scaffold implantation. After 4 weeks of implantation, M1 macrophage infiltration around the scaffold decreased markedly and M2 macrophage numbers increased markedly. The main consequence is that the early inflammatory response period is followed by a later period of tissue repair and the infiltrating macrophages are predominantly M2 anti-inflammatory macrophages, as is the case with surgical trauma and stent implantation. Our experimental results are consistent with previously reported findings. As tissue repair time passes, macrophages that infiltrate the damaged tissue progressively migrate from M1 to M2 polarization during the repair process. This, combined with the natural law of tissue repair, further demonstrates that our implanted scaffold is highly biocompatible and capable of guiding in vivo tissue regeneration [42,43].

However, there are still many unresolved challenges in implanting artificial IVD scaffolds into the natural disc space as a replacement for the original IVD, mainly including (a) the ability to withstand complex mechanical loads, (b) fixation in the disc space without positional shifts after implantation, (c) the ability to integrate well with the adjacent vertebrae, and (d) guidance of tissue to regenerate functional tissues. To overcome these challenges, we proposed as a scientific hypothesis the development of a novel BMI-SF scaffold capable of achieving regeneration of severely degenerated IVD. The above research results have tentatively demonstrated that our fabricated BMI-SF scaffold can be suitable for tissue regeneration by regulating cell growth behavior and guiding tissue infiltration. To further prove our scientific hypothesis, we implanted the BMI-SF scaffold and the control scaffold into the disc space of the rat caudal spine. In order to prevent the displacement of the BMI-SF scaffold and the control scaffold after implantation, we repaired the disc space of the segments implanted with the scaffold using PCL patches (Fig. 6A). The MRI results showed that the disc space implanted with the BMI-SF scaffold and the control scaffold could markedly restore the IVD height compared with the discectomy segment, and the MRI results at 12 weeks postoperatively showed that there were more hydrated signals in the disc space of the BMI-SF scaffold implanted segment, which was probably associated with these segments having greater soft tissue filling (Fig. 6B and C). At 4 and 12 weeks postoperatively, the anatomical observation of the rat caudal spine revealed that there was soft tissue similar to the natural IVD in the disc space of the BMI-SF scaffold and the control scaffold implantation, connecting the upper and lower vertebrae. The disc space had a specific height, whereas the disc space of the discectomy segments showed a marked loss of IVD height due to the lack of soft tissue filling and fusion between some adjacent vertebrae. The biomechanical test results of the motion segments after in vivo regeneration showed that the implanted segments of the BMI-SF scaffold had biomechanical strengths more similar to those of the natural IVD, proving that the BMI-SF scaffold was able to effectively restore the biomechanical function of the implanted segments (Fig. 6D to F). To be honest, since our scaffold material is SF, the BMI-SF scaffold fabricated by the scaffold preparation process used in this study can provide some rigid support for tissue defects in terms of mechanical properties, but it still has some deficiencies in terms of elasticity, suggesting that we can consider optimizing the manufacturing process to improve the elasticity of SF scaffolds and thus increase their application potential. The staining results of the IVD sections showed that the disc space with the BMI-SF scaffold implantation had more soft tissue filling (Fig. 7). These results indicate that the BMI-SF scaffold we developed can overcome some of the challenges currently faced in the application of artificial IVD scaffold, which supports the development of tissue-engineered IVD.

In summary, we innovatively propose the feasibility of constructing an artificial IVD scaffold using microchannel technology. The data presented in this study suggest that the BMI-SF scaffold has great repair potential in guiding natural regeneration of IVD. Of course, the regenerative ability of the BMI-SF scaffold was only investigated in this study, and it is worth investigating whether the cell-free scaffold’s ability to guide tissue regeneration can be improved by incorporating cells or bioactive substances. Additionally, due to the excessive motion of the rat caudal spine, it is important to consider whether it is suitable for constructing a total IVD replacement model. A suitable animal model will be crucial for determining the repair effect of implantation of the grafts in vivo, and therefore, suitable animal models such as a large animal model can provide more convincing support for the conclusions of the present study before advancing technology to clinical applications. In conclusion, this study shows that the BMI-SF scaffold has potential application in IVD regeneration and provides new ideas for the therapeutic options for patients with severely degenerated IVD.

In conclusion, we successfully fabricated a novel BMI-SF scaffold containing NP and AF to mimic natural IVD. The BMI-SF scaffold has excellent biocompatibility, and its microchannels can guide cell and tissue infiltration into the scaffold, thereby promoting tissue regeneration. The IVD replacement model in rats showed that the BMI-SF scaffold integrated well with the adjacent vertebrae, which helped restore the height and partial mechanical properties of the IVD. Overall, our work on novel artificial IVD based on microchannels provides a new strategy for regeneration of severely degenerated IVD.

## Data Availability

All data associated with this study are present in the paper or the Supplementary Materials.
