# Supplementary material for: Biomimetic Microchannel Integrated Silk Fibroin Scaffold for Regeneration of Intervertebral Disc Degeneration
Source: Biomater Res. 2025 May 28;29:0203. doi: 10.34133/bmr.0203 (PMC12117185; doi:10.34133/bmr.0203)
Supplement: Supplementary 1 — Figs. S1 to S3 Movies S1 and S2 [file bmr.0203.f1.zip › Figure legends of the supplementary figures.docx]

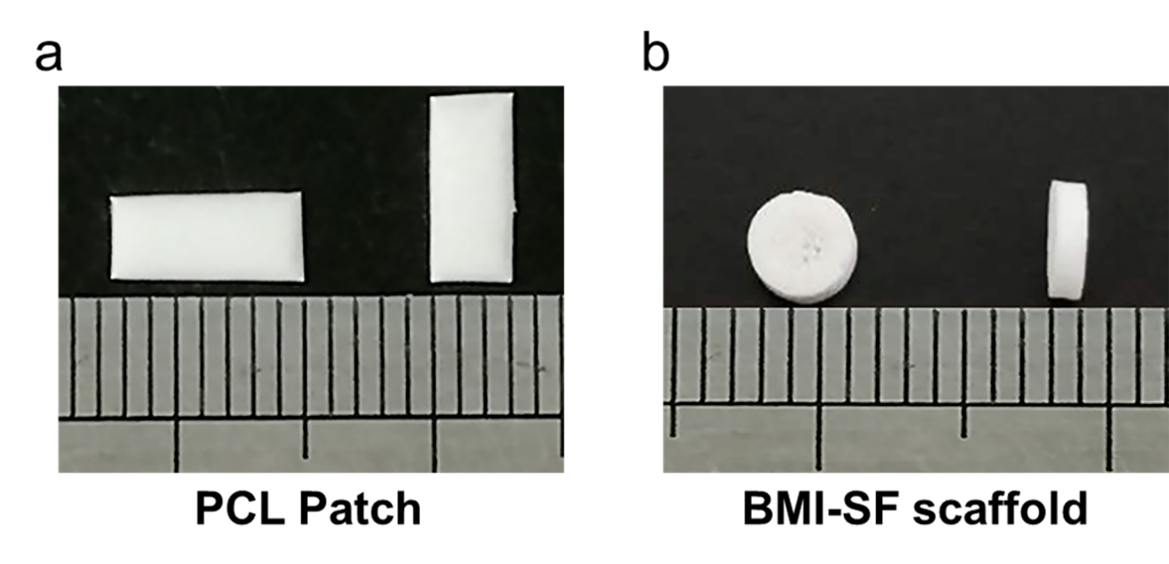
**Supplementary** **Materials**

**Fig. S1** The macroscopic morphology of the PCL patch and the BMI-SF scaffold. a) Measurement of the length and width of the PCL patch. b) Measurement of diameter and thickness of the BMI-SF scaffold.


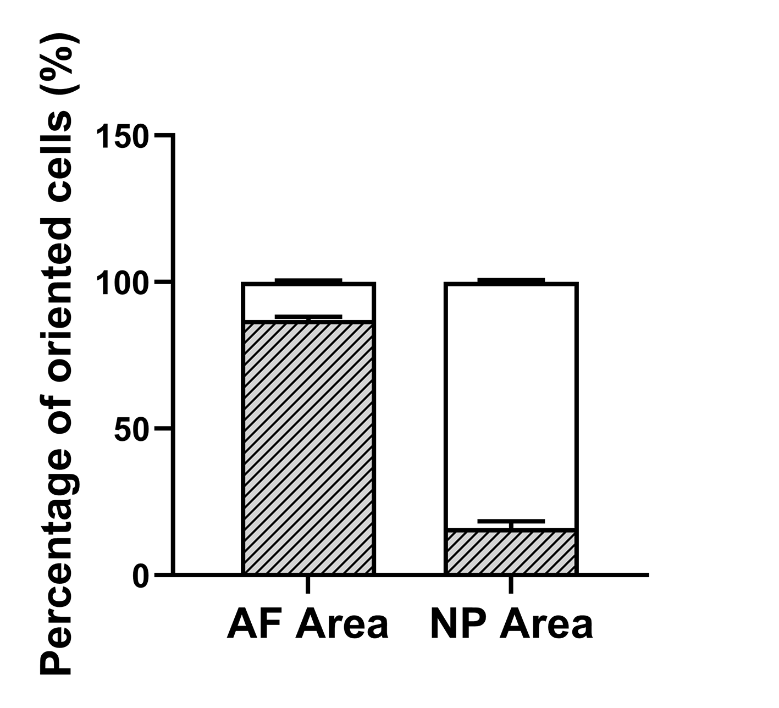


**Fig. S2** Calculation of oriented cells in the AF area and NP area of the BMI-SF scaffold. Grey area represents the proportion of orientated cells in the total cell count of the AF/NP area.


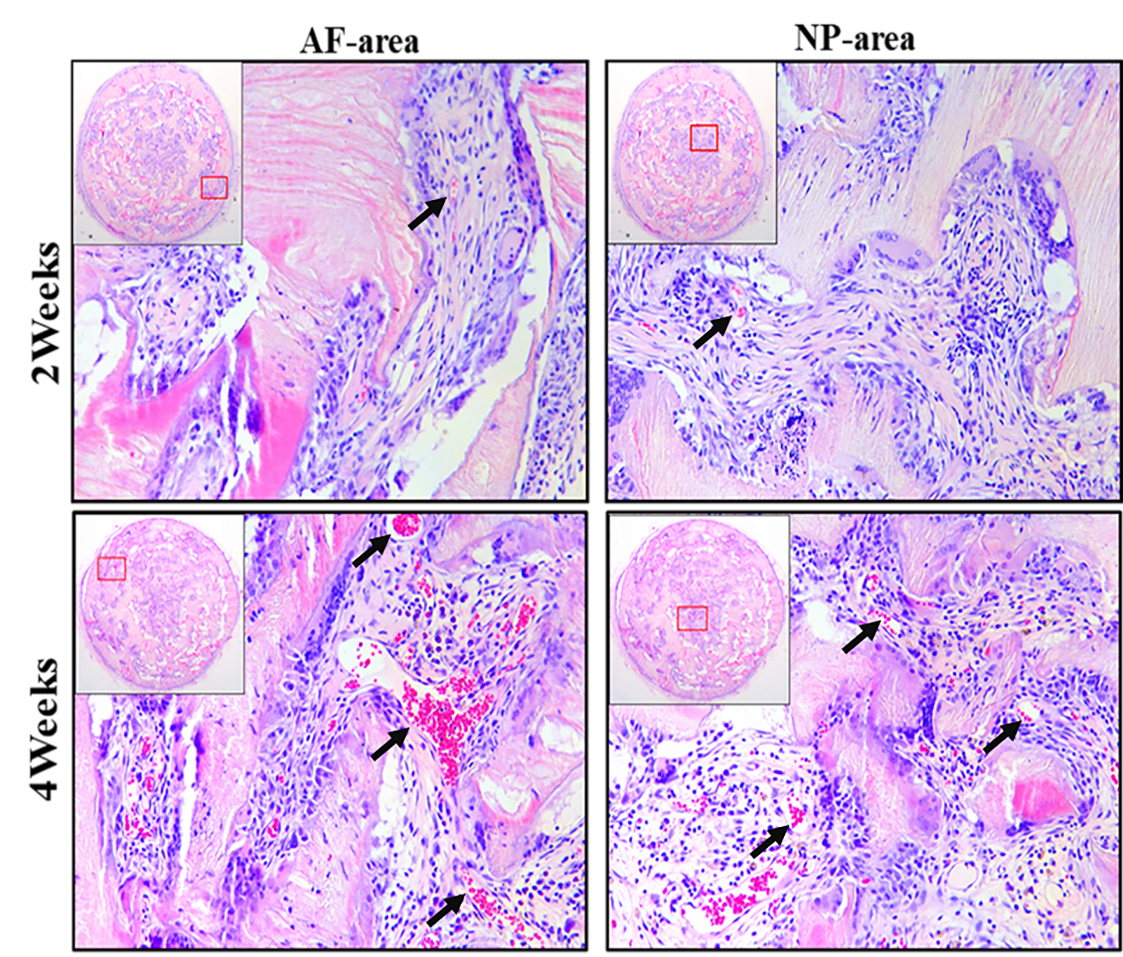


**Fig. S3** H&E staining showing cell infiltration and new vessels in the BMI-SF scaffold through microchannels at 2 and 4 weeks. Black arrows indicate the new vessels filled with erythrocytes.

**Movie 1** 3D Micro-CT reconstruction animation showing the microchannel structure inside the BMI-SF scaffold.

**Movie 2** 3D Micro-CT reconstruction animation showing the internal structure of the control scaffold.
